# Supplementary material for: Spectral Properties Echoing the Tautomerism of Milrinone and Its Application to Fe3+ Ion Sensing and Protein Staining
Source: Biosensors (Basel). 2022 Sep 20;12(10):777. doi: 10.3390/bios12100777 (PMC9599543; doi:10.3390/bios12100777)
Supplement: Supplementary file 1 [file biosensors-12-00777-s001.zip › biosensors-1915511-supplementary.pdf]

Supporting Information

Spectral Properties Echoing the Tautomerism of Milrinone and Its Application to Fe^{3+} Ion Sensing and Protein Staining

Hanming Zhu, Pan Ma, You Qian, Jiaoyun Xia, Fuchun Gong *, Lusen Chen and Lujie Xu

College of Chemistry and Chemical Engineering, Changsha University of Science and Technology, Changsha 410114, China
* Correspondence: gongfc139@163.com; Tel.: +86-0731-85258733

Table of contents

1. **Characterization of MLR…………………..………….................2-4**

2. **Effect of solvents on the fluorescence spectra of MLR…………5**

3. **Effect of pH on fluorescence of MLR……………………………6**

4. **Determination of fluorescence quantum yield……………………7**

1. Characterization of MLR

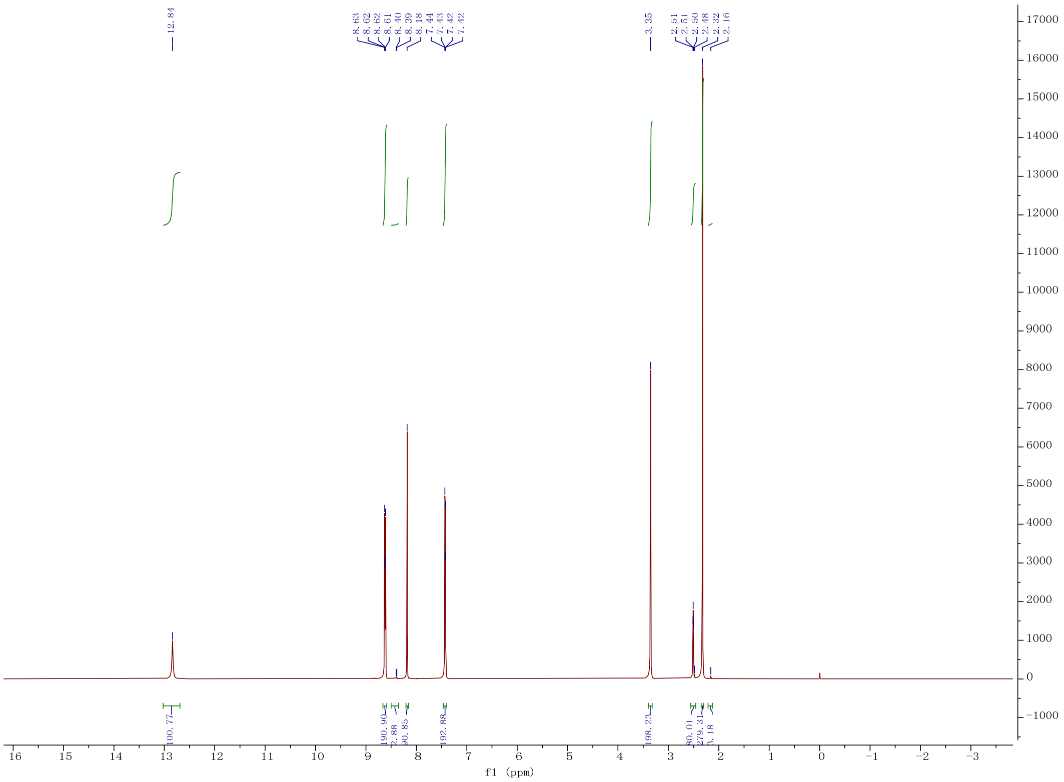

Figure S1 1H NMR of MLR

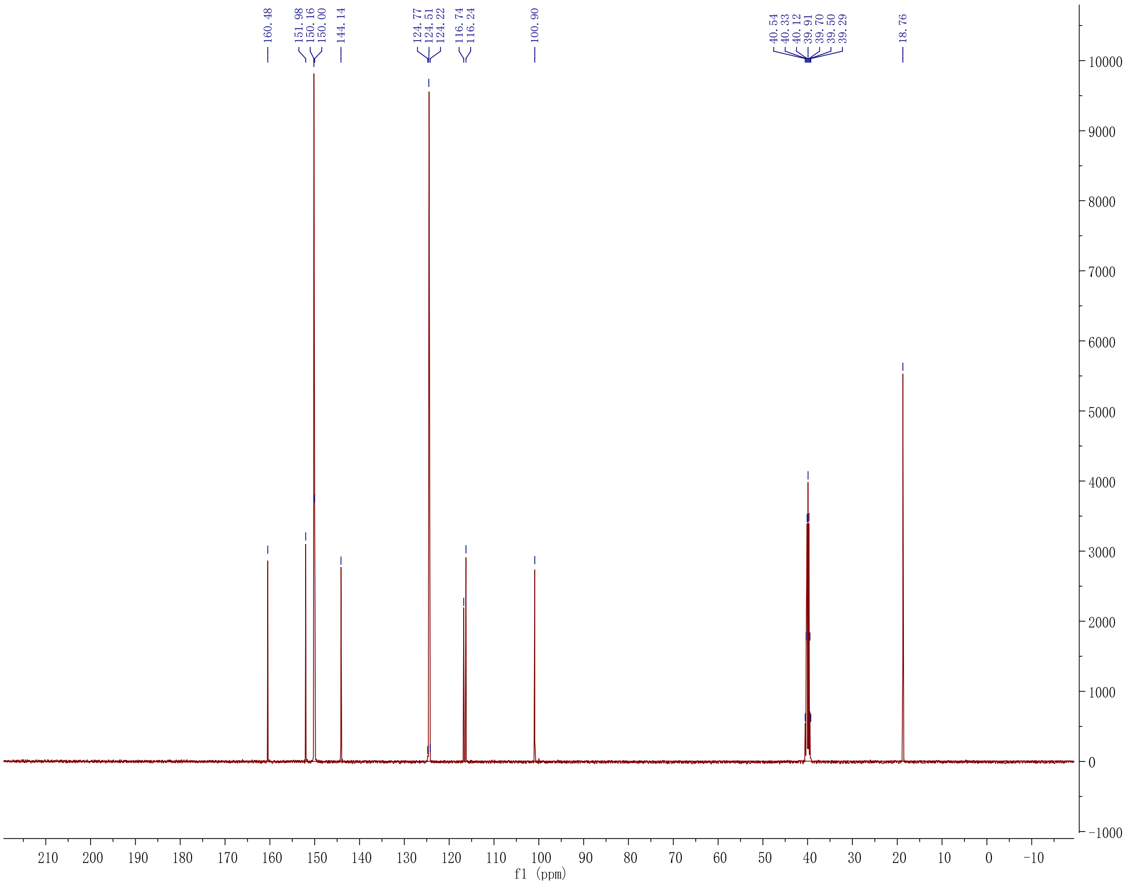

Figure S2 13C NMR of MLR

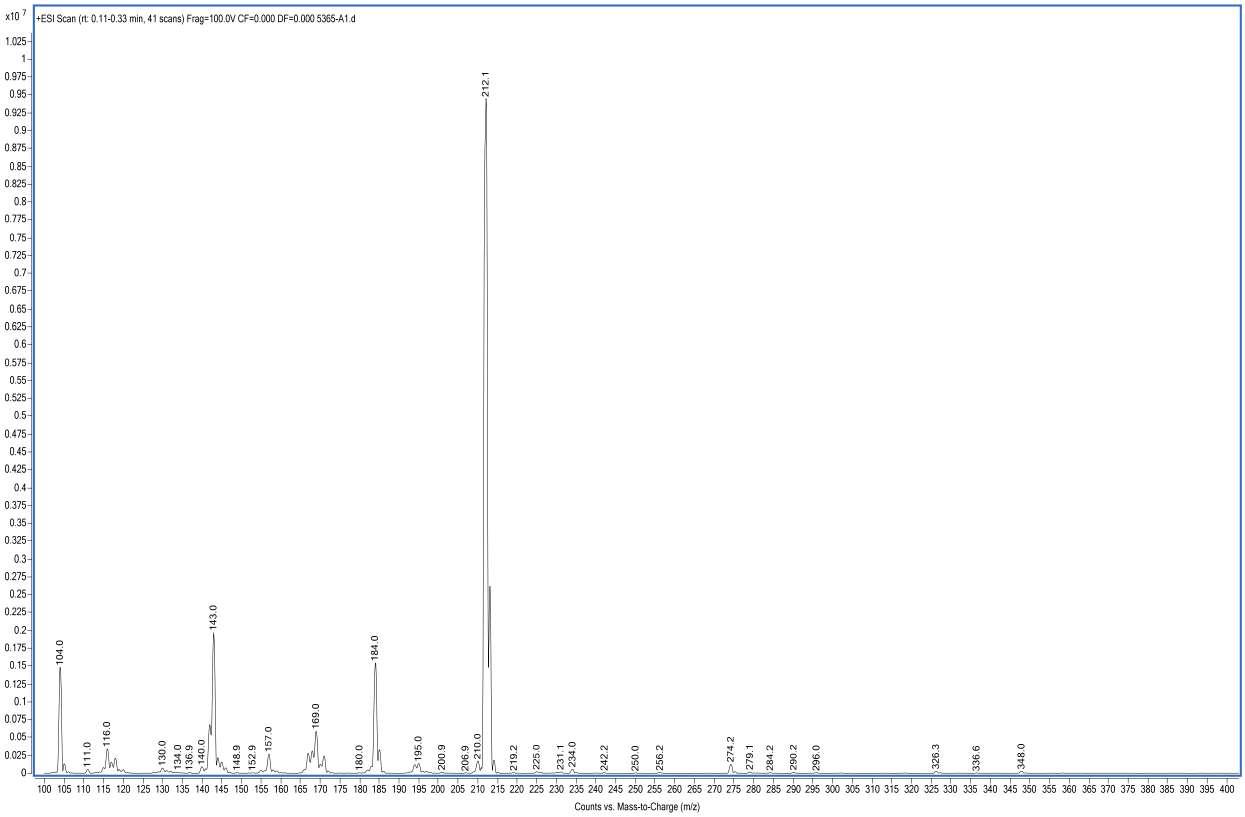

Figure S3 ESI-MS of MLR

2. Effect of solvents on the fluorescence spectra of MLR

Table S1. Determination results of Φ_f of MLR in eight different solvents

Solvents	λex/nm	λem/nm	Φ_f
Tetrahydrofuran	262,318	400	0.58
Acetonitrile	263,320	402	0.52
N,N-dimethylformamide	269,319	404	0.57
Ethanol	261,317	405	0.45
1, 4-dioxane	267,321	409	0.56
Ethylacetate	260,322	400	0.55
Cyclohexane	261,323	407	0.54
Acetic acid	263,327	402	0.36

3. Effect of pH on the fluorescence of MLR

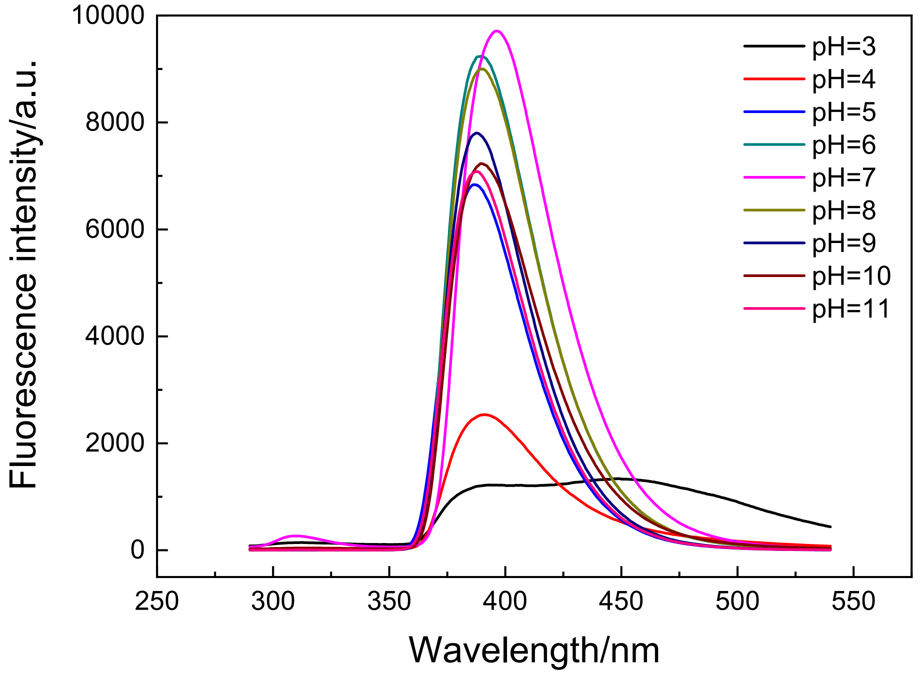

Figure S4 Effect of pH on the fluorescence of MLR

4. Determination of fluorescence quantum yield

The fluorescence quantum yield (FQY) of MLR in different solvents were determined using quinine sulfate (FQY=0.55 at 360 nm) in sulfuric acid (0.50 mol.L^{-1}, η =1.47) as the standard substance. The absolute FQY values were calculated corresponding to the following equation:

$$\Phi_u=\Phi_s(I_u/I_s)(A_s/A_u)(\eta^2{}_u/\eta^2{}_s)$$

Φ is fluorescence quantum yield; I is the measured integrated fluorescence intensity; A is the optical density measured at the selected excitation wavelength; η is the refractive index. The subscript "*s*" refers to the standard FQY of the referenced quinine sulfate. The subscript "*u*" refers to the unknown FQY of the fluorescent PNPs. In order to minimize re-absorption effect, absorbance in the 1.0 cm fluorescence cuvette was kept under 0.1 at the excitation wavelength of 360 nm.
